# Morphological and molecular characterization of a spontaneously tuberizing potato mutant: an insight into the regulatory mechanisms of tuber induction

**DOI:** 10.1186/1471-2229-8-117

**Published:** 2008-11-21

**Authors:** Lukas Fischer, Helena Lipavska, Jean-Francois Hausman, Zdenek Opatrny

**Affiliations:** 1 Department of Plant Physiology, Charles University in Prague, Faculty of Science, Vinicna 5, CZ 128 44 Prague 2, Czech Republic; 2 Department Environment and Agrobiotechnologies, Centre de Recherche Public – Gabriel Lippmann, 41, rue du Brill, L-4422 Belvaux, GD Luxembourg

## Abstract

**Background:**

Tuberization in potato (*Solanum tuberosum *L.) represents a morphogenetic transition of stolon growth to tuber formation, which is under complex environmental and endogenous regulation. In the present work, we studied the regulatory mechanisms and the role of different morphogenetic factors in a newly isolated potato mutant, which exhibited spontaneous tuberization (ST). The ST mutant was characterized in detail at morphological, physiological and biochemical levels.

**Results:**

Tuberization of the ST mutant grown in the soil was photoperiod-insensitive; predominantly sessile tubers formed directly from axillary buds even under continuous light. Single-node cuttings of the ST mutant cultured *in vitro *frequently formed tubers or basal tuber-like swellings instead of normal shoots under conditions routinely used for shoot propagation. The tuberization response of ST cuttings under light was dependent on sucrose, the concentration of which had to exceed certain threshold that inversely correlated with irradiance. Gibberellic acid prevented tuberization of ST cuttings, but failed to restore normal shoot phenotype and caused severe malformations. Carbohydrate analysis showed increased levels of both soluble sugars and starch in ST plants, with altered carbohydrate partitioning and metabolism. Comparative proteomic analysis revealed only a few differences between ST- and wild-type plants, primary amongst which seemed to be the absence of an isoform of manganese-stabilizing protein, a key subunit of photosystem II.

**Conclusion:**

ST mutant exhibits complex developmental and phenotypic modifications, with features that are typical for plants strongly induced to tuberize. These changes are likely to be related to altered regulation of photosynthesis and carbohydrate metabolism rather than impaired transduction of inhibitory gibberellin or photoperiod-based signals. The effect of gibberellins on tuberization of ST mutant suggests that gibberellins inhibit tuberization downstream of the inductive effects of sucrose and other positive factors.

## Background

Tuber induction in potato (*Solanum tuberosum *L.) is a complex, multilevel process, which integrates environmental and internal signals to ensure optimal life strategy during the growing season (reviewed in [34,37]). Environmental requirements for tuberization vary among potato subspecies and varieties. *S. tuberosum *ssp. *andigena *strictly requires short-day photoperiod for tuber formation, however, *andigena *plants with inactivated phytochrome B gene (involved in photoperiod sensing) tuberize even under continuous light [17]. *S. tuberosum *ssp. *tuberosum *plants are less dependent on day-length. Generally, photoperiodic signal is integrated with other environmental factors, such as nitrogen availability, temperature and light intensity, as well as with the overall metabolic status of the plant, so that the plants can tuberize even under long-day photoperiods (reviewed in [16]).

Plants exposed to conditions that favor tuberization display significant metabolic and growth changes in both above- and underground organs. Soon after transfer to inducing conditions, the rate of photosynthetic assimilation, starch synthesis and sucrose export from leaves substantially increase [25]. Underground stolons of induced plants stop elongating and begin to swell, eventually forming tubers [47]. Active storage of carbohydrates in tubers leads to reduced vegetative growth, flowering and fruiting (reviewed in [7]).

In addition to stolons, practically any axillary bud on stems or stem cuttings can form a tuber, provided the plant has been induced to tuberize. Depending on the degree of induction, buried axillary buds on single-node cuttings cultured *in vivo *form tubers, either sessile ones or attached to stolon tips (reviewed in [7]). Single-node cuttings tuberize synchronously when cultured *in vitro *in darkness on a medium with reduced nitrogen content and optimal sucrose concentration [15,48]. However, only cuttings taken from induced plants develop tubers directly. Cuttings from non-induced plants form primarily long shoots/stolons instead of tubers irrespective of the sucrose concentration in the medium [11].

Tuber initiation, either on intact plants or single-node cuttings, was demonstrated to be under the coordinated control of many plant hormones including gibberellins, cytokinins, abscisic acid, jasmonic acid and others. Although the evidence for involvement of some hormones remains controversial (e.g. [38]), the role of gibberellins as key negative regulators has been established as unequivocal [6-8,34]. Exogenous application of gibberellins promoted stolon elongation and inhibited tuber formation, whereas a drop in gibberellin level preceded the first visible signs of swelling in stolon apices [48]. Besides being dominant regulators of stolon-to-tuber transition, gibberellins also play a role in the photoperiodic control of tuberization. Reduced gibberellin levels were shown to accompany changes in morphology, metabolism and gene expression in leaves of plants induced to tuberize [27,6,1,34]. However, while photoperiod sensing and gibberellin signaling are interconnected in many respects [1], they are considered to inhibit tuberization at least partially through independent pathways [28].

In the present work, we have characterized a novel mutant of potato (*Solanum tuberosum *L. ssp. *tuberosum*), which displays a strong tendency to **s**pontaneous **t**uberization (ST) under both *in-vitro *and *in-vivo *conditions. The phenotype of the ST mutant has been analyzed in detail at the morphological, biochemical and molecular levels. Because the number of available potato mutants or genetically modified lines with altered tuberization is limited, the ST mutant provides a rare and useful tool for studying various aspects of tuber induction. Based on the characterization of this ST mutant, we propose a revised model of the regulatory roles of sucrose and gibberellins in the inductive mechanism of potato tuberization.

## Results

### Molecular characterization of ST mutant

**Figure 1:**
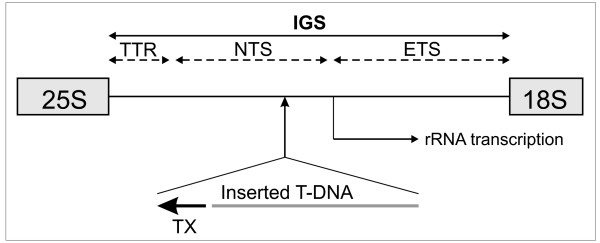
**Scheme of the T-DNA insertion into non-coding region between 25S and 18S rDNA genes in ST mutant**. TX, modified CaMV 35S promoter for gene activation; IGS, intergenic spacer; TTR, transcription terminator region; NTS, non-transcribed spacer; ETS, external transcribed spacer (the scheme of the intergenic spacer according to [46]).

The ST mutant originates from cv. Lada plants that were transformed with a gene-trap construct for random gene activation (Perry and Hrouda, unpublished results). We have identified a single T-DNA insertion in the ST mutant genome by Southern hybridization using probes prepared from both ends of the T-DNA (data not shown). The adjacent chromosomal DNA, isolated by a plasmid rescue procedure, was shown to share more than 90% identity with a database sequence annotated as rDNA intergenic spacer (accession number X65489; [4] Figure 1). The transcription of this sequence did not differ substantially between cv. Lada and the ST mutant as determined by Northern hybridization of total leaf RNA (data not shown).

**Figure 2:**
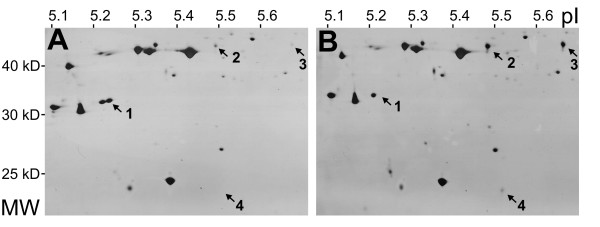
**Leaf proteomes of Lada and ST plants**. Total proteins from (A) cv. Lada and (B) ST mutant were separated by isoelectric focusing (pH 5–6) and SDS-PAGE. Arrows indicate protein spots differing between the lines: (1) manganese-stabilizing protein isoform; (2,3) patatins; (4) cysteine protease inhibitor. MW, molecular weight; pI, isoelectric point.

Proteomic analysis revealed only few organ-specific differences between the corresponding organs (leaves, stems and roots) of Lada and ST plants. A single protein spot (spot 1 in Figure 2) was completely missing in ST leaves, while three others (spots 2, 3 and 4 in Figure 2) were present at substantially higher levels. MALDI-TOF MS analysis identified the up-regulated protein spots 2 and 3 as a new member of the patatin family (the two spots probably representing two forms of the same protein), and the up-regulated spot 4 as an inhibitor of cysteine proteases. The levels of these two up-regulated proteins responded positively to increased sugar supplementation in both the ST mutant and wild-type plants (data not shown). The missing protein spot 1 was identified as an isoform of manganese stabilizing protein (MSP) of photosystem II. This protein was consistently absent in leaves from ST plants grown under any of the conditions tested in this study (on media with 1%, 3% or 5% sucrose, and in the greenhouse). By contrast, the same MSP isoform was consistently present at the same level in leaves from Lada plants grown under any of the test conditions.

### Morphological characterization of ST mutant

#### *In vitro*

**Figure 3:**
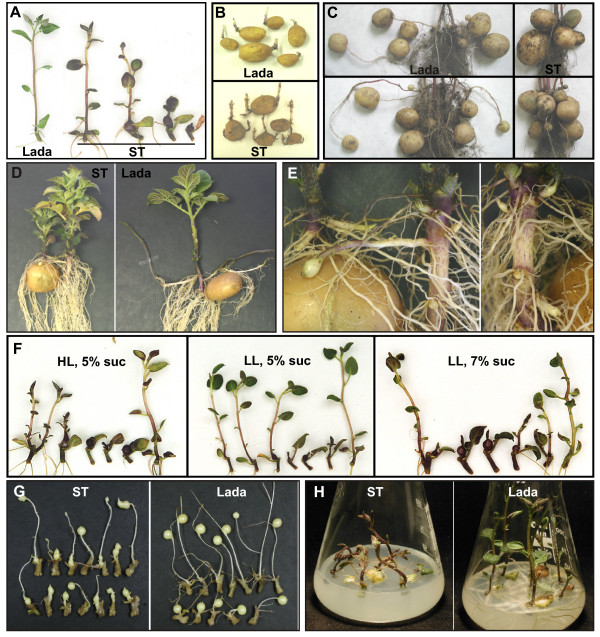
**Morphology of Lada and ST plants**. (A) Four-week-old plants grown *in vitro *under standard culture conditions (3% sucrose); (B) Sprouting of tubers stored for six months at 4°C; (C) Arrangement of tubers on three-month-old plants cultivated in pots under 15/9 (L/D) photoperiod; (D) Phenotype of four-week-old plants grown from tubers in pots under continuous light; (E) Detailed view on tuberization of ST plant grown under continuous light; note swelling buds, which are going to form sessile tubers; (F) Single-node cuttings of ST plants cultured *in vitro *on media with variable levels of sucrose (5% or 7% suc) under high (HL) or low (LL) irradiance (PPFD of 400–500 or 50–60 μmol m^-2 ^s^-1^, respectively); cuttings are arranged from the most basal segment on the left to the apical one on the right; (G) Tuberization of single-node cuttings cultured on the tuberization medium in darkness for five weeks; (H) Three-week-old plants grown in the light on the medium supplemented with 5% sucrose and 3 mg/l GA_3_.

The ST mutant was selected on the basis of its extraordinary phenotype – frequent spontaneous tuberization of single-node cuttings under non-inducing conditions, which were routinely used for the maintenance of shoot cultures *in vitro*. The growth of ST plants *in vitro *depended on the original position of the cutting used for propagation. Cuttings excised from the middle or the subapical part of ST plants predominantly developed either green-purple tubers (directly from the axillary buds) or modified shoots with basal tuber-like swellings, with both the shoot and the swelling growing simultaneously (Figure 3A). In contrast, cuttings taken from the base of ST plants gave rise to nearly normal shoots. They differed from Lada shoots only in higher content of anthocyanins, reduced rooting and slightly more compact growth habit (Figure 3A). During prolonged cultivation for more than four weeks, ST plants regularly formed multiple branches at the base, while Lada plants branched from subapical nodes. The ST mutant did not show any usual symptoms of senescence and remained dark-green for two to three months, in contrast to Lada plants that became yellowish and senescent several weeks earlier.

#### *In vivo*

**Figure 4:**
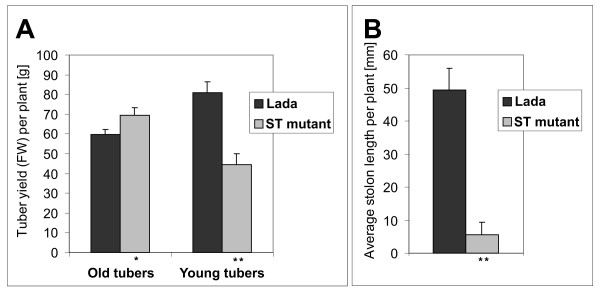
**Tuber yield and stolon length of Lada and ST plants growing in soil under long days**. (A) Tuber yield per plant; (B) Average stolon length per plant (plants grown from young tubers). The histograms represent means +SD from 5 replicates per variant; * and ** = significantly different from the corresponding organs of control (Lada) at 0.01 ≤ *P *< 0.05 and *P *< 0.01, respectively, according to Student's paired t-test.

Shoots of ST plants growing from tubers in the soil under long-days were slightly more compact compared with Lada plants. Fully developed leaves of ST plants started to be chlorotic, in contrast to the dark-green leaves seen in the *in-vitro *plants described above. However, the soil-grown plants formed ordinary tubers instead of tuber-like swellings. The growth of both ST and Lada plants strongly depended on the age of the mother-tuber, which influenced the number of tuber sprouts and shoot branches. Young ST tubers stored for one month and Lada tubers formed only single or few sprouts, in contrast to multiple sprouting of old ST tubers stored for 6 to 8 months (Figure 3B). However, ST mutants and Lada shoots derived from old tubers remained branchless, while Lada plants grown from young tubers branched vigorously. The yield of tubers, correlating with the overall growth, was almost twice higher in these young-tuber-derived Lada plants as compared to branchless young-tuber-derived ST plants. With old mother-tubers, the overall growth and the yield were more or less comparable between the two lines (Figure 4A).

Tubers on Lada plants were formed on stolons of variable length, whereas ST tubers were massed around underground shoots due to very short or no stolon formation (Figure 3C). The average Lada stolon was about ten times longer than that of ST plants (Figure 4B).

The strong tendency of ST plants to form tubers was evident even under continuous light (Figure 3D). The first tubers formed on very short stolons after four weeks' planting of the mother tubers. Tubers developing later were predominantly sessile, arising directly from underground axillary buds (Figure 3E). In contrast, Lada plants cultivated under the same conditions developed tubers on very long stolons (Figure 3D) more than four weeks later. ST plants cultivated under continuous light developed flower buds approximately at the same time as tubers; no flower bud formation was observed in Lada plants.

### Factors affecting tuberization *in vitro*

#### Sucrose and light

As mentioned above, single-node cuttings taken from ST plants frequently tuberized when cultured *in vitro *on a standard propagation medium (3% sucrose) and long-day photoperiod. Increasing sucrose concentration (5%) moderately raised the frequency of tuberization (Table 1), whereas a slight decrease in sucrose concentration (2.5%) resulted in a marked decline in tuberization frequency (Table 1). The formation of tubers and tuber-like swellings was almost completely inhibited when cuttings were pre-cultured on the low-sucrose (2.5%) medium for two or more days before transfer to the high-sucrose (5%) medium. The growth of shoots on the pre-cultured cuttings was retarded and the shoots were chlorotic (data not shown).

**Table 1:** Tuberization of the ST mutant under various conditions *in vitro*

	Normal shoots	Tuber-like swellings	Tubers
High irradiance (PPFD 400–500 μmol m^-2 ^s^-1^)			

1% sucrose	93%	7%	0%

2.5% sucrose	88%	12%	0%

3% sucrose	14%	67%	19%

5% sucrose	0%	36%	64%

Low irradiance (PPFD 50–60 μmol m^-2 ^s^-1^)			

3% sucrose	94%	6%	0%

5% sucrose	46%	32%	22%

7% sucrose	8%	38%	54%

Cultures were raised from single-node cuttings. Percentage values are based on 50 and 100 cuttings cultured under low and high irradiance, respectively. Lada cuttings only rarely (< 5% cuttings) formed tubers in the light under any conditions tested in this study.

The size and number of tubers and the formation of tuber-like swellings were strongly reduced when cuttings from ST plants were grown on standard medium (3% sucrose) under low irradiance (50–60 μmol m^-2 ^s^-1^; Table 1). This negative effect could be compensated by elevated sucrose concentration in the medium to 5 or 7% (Table 1, Figure 3F). In contrast to ST mutant, Lada cuttings rarely (< 5% cuttings) formed tubers in the light under any conditions tested in this study.

Under conditions commonly used for tuberization *in vitro *in the dark, cuttings from ST plants started to form microtubers synchronously on the fifth day of culture. Interestingly, the majority (70–80%) of these microtubers began to grow again from their apical meristems, forming sprouts/stolons that often developed secondary tubers (Figure 3G). In comparison, Lada cuttings started to form tubers on long etiolated shoots/stolons asynchronously after two weeks' culture, but they ceased to grow further (Figure 3G).

#### Gibberellic acid

Addition of GA_3 _(3 mg/l) to the high-sucrose medium (5%) completely prevented tuberization of ST cuttings in the light. Interestingly, the shoots grown under these conditions were severely malformed; the shoots were short, curved, purple colored and with small, folded leaves. In contrast, Lada cuttings under the same conditions formed normal shoots with slightly more elongated internodes (Figure 3H). On low-sucrose medium (2.5%) supplemented with GA_3_, both ST and Lada cuttings developed normal shoots with slightly longer internodes (data not shown).

### Carbohydrate content and dynamics

**Figure 5:**
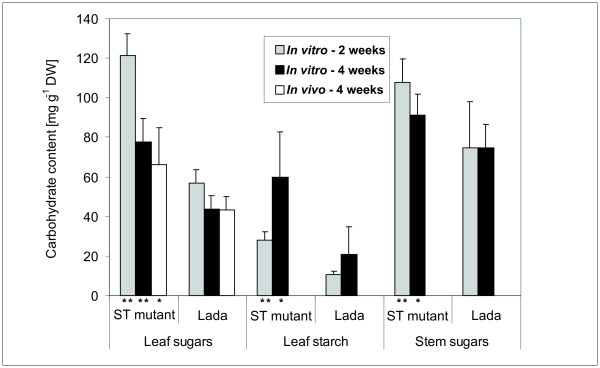
**Carbohydrate content in leaves and stems of Lada and ST plants**. Plants were grown *in vitro *from apical segments on a medium with 3% sucrose for two and four weeks and in the greenhouse (*in vivo*) for 4 weeks. The sugar contents represent totals of glucose, fructose and sucrose contents, the starch contents are expressed as glucose equivalents; the histograms represent the mean +SD from 3 to 6 replicates per variant; * and ** = significantly different from the corresponding organs of control (Lada) at 0.01 ≤ *P *< 0.05 and *P *< 0.01, respectively, according to Student's paired t-test.

The carbohydrate contents were determined in leaves and stems of ST and Lada plants grown *in vitro *on standard medium (3% sucrose) for two and four weeks, and in leaves from plants cultivated in the greenhouse. The total amounts of sugars (sucrose, glucose and fructose) and starch were constantly higher in ST plants than in Lada plants. The difference was most significant in leaves, where it was about twice higher in ST than in Lada plants (Figure 5). However, the relative proportions of sucrose, glucose and fructose did not differ markedly between corresponding organs of ST and Lada plants (data not shown).

**Figure 6:**
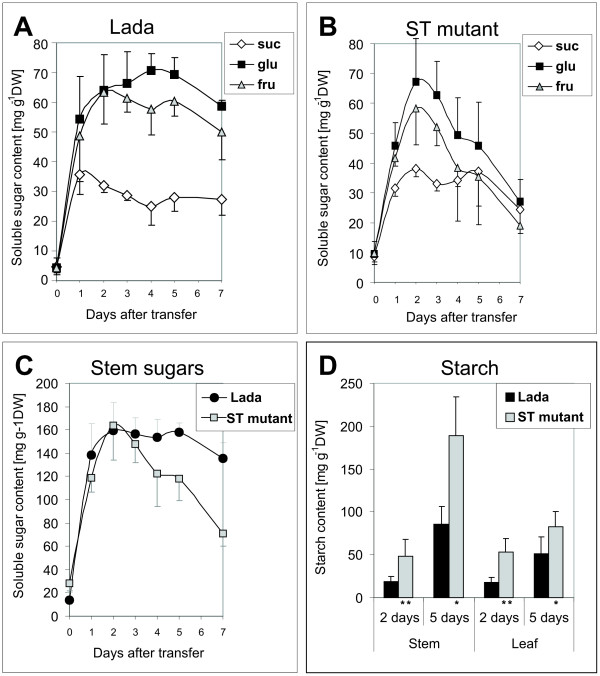
**Dynamics of sugars and starch contents**. Three-week-old *in-vitro *plants were transferred onto a liquid medium with 5% sucrose and further cultured for seven days. (A, B) Sucrose, glucose and fructose contents in stems of (A) Lada and (B) ST plants; (C) Total soluble sugar contents in stems of Lada and ST plants; (D) Starch contents in stems and leaves of Lada and ST plants two and five days after the transfer; the histograms represent the mean +SD from 3 to 6 replicates per sample; suc, sucrose; glu, glucose; fru, fructose; * and ** = significantly different from the corresponding organs of control (Lada) at 0.01 ≤ *P *< 0.05 and *P *< 0.01, respectively, according to Student's paired t-test.

In order to evaluate changes in sugar partitioning and metabolism in the ST mutant, three-week-old plants grown *in vitro *were transferred to a liquid medium supplemented with 5% sucrose to provide abundant and easily available source of sugar. Although sugar content was initially high in both Lada and ST plants, its level decreased earlier and faster in ST stems due to a decline in glucose and fructose levels (Figure 6A–C). Decreasing hexose levels correlated with enhanced starch synthesis in stems of ST plants (Figure 6D). In leaves alone, however, the levels in soluble sugar content did not differ significantly between Lada and ST plants (data not shown).

To test whether tuberization of ST mutant in the light could result from increased levels of endogenous sugars, Lada cuttings from the previous experiment were cultured on high-sucrose medium (5%) in the light. In spite of the high sugar content in the source Lada plants, the cuttings invariably gave rise to normal shoots (data not shown).

## Discussion

### ST mutant lacks the manganese-stabilizing protein isoform

The ST-mutant phenotype was initially thought to have resulted from an activating insertion of the T-DNA near some tuberization-related gene. The insertion could also cause gene inactivation if the insertion site involves a protein-coding sequence [20]. However, molecular analysis of the mutant did not support any of these possibilities, because the single T-DNA copy was found to be inserted into a repetitive non-coding rDNA intergenic region (Figure 1). As no other copy of T-DNA was detected, the genetic cause of the phenotypic changes requires further analysis. The long-term stability of the mutant phenotype indicated a mutation associated with either somaclonal variation through regeneration from dedifferentiated somatic cells [14] or transclonal variation inherent in the transformation process itself [33].

Patatins and protease inhibitors, whose representatives were shown to be up-regulated in the ST mutant as revealed by proteomic analysis, represent protein families that respond to high sucrose concentration and hyper-accumulate during tuber formation [15,22]. Both of these identified proteins were positively correlated with sugar levels in the medium, but they were specifically expressed in leaves (unpublished observations). Their high levels in the ST mutant were probably associated with higher sugar levels detected in these plants. In contrast, the absence of the MSP isoform was a stable change detected under variable conditions (and also confirmed at the nucleic acid level; unpublished observation). Possible interrelationships between this proteomic change and the phenotypic changes observed in the ST plants are discussed below in the context of detailed physiological and biochemical characterization.

### ST-mutant axillary buds can tuberize even in the light

As cv. Lada belongs to subspecies *tuberosum*, its tuberization is not strictly dependent on short-day photoperiod; tubers can be induced even under long-days if the plants' energetic balance favors the storage of assimilates [34]. Tuberization of single-node cuttings, however, is inhibited by light [7,11]. The induced state of ST plants appeared to be so strong that their cuttings tuberized even in the light. Normal shoots were formed only by the most basal cuttings that formed lateral branches over prolonged culture *in vitro*, and those pre-cultured on low-sucrose medium (2.5%). It appears that in both of these cases axillary buds were released from correlative inhibition [23] under non-inducing conditions. This indicates that the fate of axillary buds is not pre-determined, and their development to shoots or tubers depends on conditions under which they are released from the correlative inhibition. The formation of modified shoots with tuber-like swellings represents a unique, intermediate developmental program between shoot and tuber formation. It also suggests that radial cell divisions are not necessarily negatively interrelated with the cessation of the apical meristem activity during tuberization of wild-type plants as documented by [49] and [47].

### Is sucrose the last missing component of tuber-inducing signal in the ST mutant?

By studying *in-vitro *tuberization on wild-type single-node cuttings in the dark, [48] demonstrated that the frequency of tuber formation, stolon length and tuber weight was directly proportional to sucrose concentration. In the ST mutant, in contrast, sucrose affected tuberization in a stepwise manner. Only a marginal increment from 2.5 to 3% markedly increased tuberization frequency from12 to 86%, indicating that the developmental switch between shoot and tuber formation requires certain threshold concentration of sucrose (dependent on irradiance).

The role of sucrose in tuber induction is complex because it not only serves as an important source of energy, but also directly regulates the expression of both metabolic and regulatory genes involved in tuber formation (e.g. [31]). Sucrose is therefore generally regarded to be a component of tuber-inducing signals, as also demonstrated in our ST mutant, although it is not usually the limiting factor of tuberization (reviewed by [7,45]). In the ST mutant, however, sucrose probably represents the last missing component of the cascade of tuber-inducing signal in otherwise pre-induced axillary buds.

### Enhanced tuberization in ST mutant is unlikely to be associated with gibberellin or photoperiod signaling

The enhanced tuberization capacity of the ST mutant (pre-induced state) may result either from increased effects of positive regulators or reduced effects of tuberization inhibitors (gibberellins and photoperiodic signals). Tuberization of ST plants in soil, characterized by suppressed stolons formation, resembled transgenic *andigena *plants with reduced level of phytochrome B (phyB), which also appeared to be in a permanently induced state to tuberize [17]. However, despite comparable tuberization efficiency, elongated and thinner stems and light-green leaves of phyB-antisense plants [18] strongly contrasted with the phenotype of ST plants. Therefore, disturbed phyB signaling is unlikely to be responsible for the phenotypic modifications in ST plants.

High tuberization capacity of the ST mutant may also be assumed to be the result of alleviated gibberellin-mediated inhibition (reviewed in [34]). Insensitivity of the ST mutant to gibberellins can be ruled out since exogenous application of GA_3 _completely prevented tuberization. If lower levels of gibberellins were the reason, gibberellin application would have not only inhibited spontaneous tuberization, but also restored wild-type shoot morphology. Instead, the gibberellin treatment severely retarded the growth of shoots on the ST cuttings and the shoots were malformed (Figure 3H). Taken together, the enhanced tuberization capacity of the ST mutant does not seem to be directly related to either gibberellin or photoperiodic signals, indicating the existence of another mechanism that results in a strong induction of tuberization.

### Gibberellins inhibit tuberization downstream of the inducing effects of sucrose and other positive regulators

The unexpected phenotype of ST shoots on high-sucrose medium (5%) supplemented with GA_3 _suggested that although gibberellins were capable of inhibiting tuber formation they failed to repress the putative metabolic/structural changes associated with the induced state to tuberize. Similar response was described for phyB-antisense plants, which, under exposure to gibberellin treatment, formed slightly swollen stolons metabolically equivalent to tubers [18]. This indicates that the site of gibberellin action lies most downstream in the mechanism of tuber induction. This proposed model is in agreement with recent studies, which show that active GA2 oxidase-mediated degradation of bioactive gibberellins occurs in stolons prior to tuberization [19] and that gibberellin synthesis is repressed by a pair of tuberization-related homeotic transcription factors StBEL5 and POTH1 [2,5].

### Are the complex phenotypic changes in ST mutant associated with altered carbohydrate metabolism and the absence of MSP isoform?

Carbohydrates, while serving primarily nutritional function, appear to be involved in many signaling pathways regulating plant growth (reviewed in [9]). In this context, at least some of the developmental, phenotypic and proteomic alterations in the ST mutant could be readily interpreted as a direct or indirect consequence of increased levels or modified partitioning of carbohydrates. In addition, carbohydrate metabolism is closely interconnected with cytokinin signaling. Therefore, the mutant features such as delayed senescence, basal branching, and reduced rooting, which may be related to cytokinin regulation (reviewed in [29]), could also result from elevated carbohydrate levels [35]. However, increased sugar levels in shoots following culturing on high-sucrose medium were not enough to mimic ST mutant-like tuberization in the wild-type cv. Lada. This suggests that enhanced carbohydrate levels concomitant with enhanced tuberization are probably secondary effects of superordinary changes either in carbohydrate metabolism and transport or redox signaling [37], both of which have generally stronger impact on plant growth regulation compared with simple carbohydrate levels [9].

What could be the connection between altered carbohydrate metabolism (or redox signaling) and the absence of a MSP isoform in the ST mutant? MSP represents a key subunit of oxygen-evolving complex of photosystem II, which is located at the start of the photosynthetic electron transport chain. Thus the MSP has a great potential to affect the rate of photosynthesis and consequently chloroplast redox state and carbohydrate metabolism [30,42]. In *Arabidopsis *there are two MSP isoforms with substantially different functions [30]. Besides stabilizing manganese, MSP isoforms were found to possess GTPase and carbonic anhydrase activities. MSPs are involved not only in water splitting and oxygen evolution, but also in other processes, such as replacement of photo-damaged D1 protein in the reaction center of photosystem II ([26] reviewed in [42]). In green algae, MSP protein was also shown to have a thioredoxin-like activity, which is theoretically directly involved in the regulation of enzyme activity in the Calvin cycle [13]. In higher plants, it is assumed that a large pool of free, assembly-competent MSP proteins in thylacoid lumen [12] allows fine-tuning of PSII activity through exchanges between different PSII-bound MSP isoforms [42].

*Arabidopsis *mutants lacking either of the two MSP isoforms remained photosynthetically competent, but were moderately retarded in growth. The mutants differed in their photosynthetic parameters: plants lacking *MSP1 *gene (*psbo1 *line) had reduced PSII activity, while those lacking *MSP2 *gene (*psbo2 *line) had the PSII activity unexpectedly increased [26]. A similar increase in PSII activity, twice higher than in cv. Lada, was also detected in the ST mutant (Fischer and Hola, unpublished observation), indicating a role of the missing MSP isoform in generating the ST mutant phenotype. Consequently, the down-regulated photosynthesis in the ST mutant could be responsible for altered redox signaling, carbohydrate metabolism and other phenotypic changes.

## Conclusion

The unique phenotype of the ST mutant appears to be associated primarily with carbohydrate metabolism rather than with gibberellin or photoperiod signaling, unlike the majority of existing potato mutants and transgenics with enhanced tuberization. The absence of MSP isoform in the ST mutant suggests that the phenotypic changes may have resulted from altered photosynthetic machinery, which is of high general interest. By analyzing the effects of sucrose and gibberellins, the key regulators of tuberization, this study clearly demonstrated that they affect tuberization at different steps of the inductive mechanism. In contrast to the classical view, gibberellins appear to regulate tuberization at a site most downstream of the induction pathway(s). Although capable of inhibiting tuber formation, exogenous gibberellin failed to revert presumptive functional changes associated with the permanently induced tuberizing state. Sucrose above a threshold level apparently has the capacity to regulate the transition of the light-induced growth of axillary buds from shoots to tubers. The formation of modified shoots with tuber-like swellings indicates that tuber formation is not necessarily negatively interconnected with cessation of the apical meristematic activity.

## Methods

### Plant material

The ST mutant was selected from a population of potato (*Solanum tuberosum *L.) cv. Lada (breeder Selekta Pacov, Plc., Czech Republic) transformed with a gene-trap construct (Krizkova and Hrouda, unpublished results). The binary vector, pPCV631-L-TX [21], used for *Agrobacterium tumefaciens*-mediated transformation, was constructed from the vector pPCV631 [20]. The *TX *promoter was inserted into pPCV631, next to the left border of the T-DNA, in order to induce transcription of a neighboring gene at the site of insertion [21]. The T-DNA further carried neomycin and hygromycin phosphotransferase genes for selection of transformed plant cells and bacterial sequences (see bellow) to allow isolation of the flanking plant DNA by the plasmid rescue procedure.

### Experimental conditions

Plants were propagated *in vitro *using single-node cuttings (with a leaf subtending) taken from four-week-old stock cultures. For ST mutant, apical segments were exclusively used to obtain normal shoots without tuberization. The plants were cultured in 100 ml Erlenmayer flasks (covered with aluminum foil) under a 16 h photoperiod (or continuous light) with PPFD approximately 400–500 μmol m^-2 ^s^-1 ^(daylight fluorescent tubes; Osram, Wintherthur, Switzerland) on semisolid LS medium [24] containing 3% sucrose. Conditions of individual treatments are indicated in the 'Results' section (Table 1). All additives, including growth regulators (GA_3_, BAP; Sigma-Aldrich, St. Louis, USA), were added prior to sterilization by autoclaving at 121°C for 20 min.

Tuberization frequency of Lada and ST mutant plants in the light was evaluated under the same conditions used for shoot propagation with variable levels of sucrose (1%, 2.5% = low sucrose, 3% = standard, 5% = high sucrose and 7%) and PPFD (50–60 and 400–500 μmol m^-2 ^s^-1^). Single-node cuttings, excluding the most basal ones, were taken from four-week-old plants. Alternatively, cuttings were taken from three-week-old plants, which were pre-cultured in a liquid medium containing 5% sucrose for three days to increase the level of endogenous sugars. Standard tuberization of leafless single-node cuttings was performed in the dark in Magenta vessels on a medium containing 7% sucrose, ten-times reduced inorganic nitrogen and 2.0 mg/l BAP, according to [15]

Plants cultivated in the soil were grown from tubers of variable age (stored at 4°C for one or 6 to 8 months) in 5 to 10 liter pots under a 15 h photoperiod (natural illumination) or under continuous light (PPFD approximately 800 μmol m^-2 ^s^-1^) for 2 to 3 months. Tuber yield was evaluated after about three month.

### Genetic analysis

Southern and Northern hybridization: total DNA and RNA were isolated from leaves of *in vitro-*grown plants according to [39] and [41], respectively. Blotting was performed as described by [36]. Hybridization with probes labeled by DIG-dUTP (Roche Molecular Systems, Inc., Mannheim, Germany) was done according to the manufacturer's instructions.

A plasmid rescue procedure was modified from [20]. The total DNA was cleaved by EcoRI, yielding 2.5 kbp-long fragment (detected by Southern hybridization) of plant DNA attached to the left part of the T-DNA containing the *TX *promoter, the bacterial origin of replication and ampicillin resistance gene. Diluted restriction fragments (15 μg ml^-1^) were circularized with T4 DNA ligase and used for electroporation of ultracompetent *E. coli *strain DH10B tolerant to methylated DNA (Invitrogene, San Diego, USA). The plasmid DNA was isolated using SV Minipreps DNA purification system (Promega, Madison, USA) and analyzed by restriction and sequencing.

### Proteomic analysis

The total protein extraction procedure and the two-dimensional analysis were carried out as described by [10], with modifications introduced by [32]. Proteins were isolated from 250 mg (fresh weight) of (a) leaves, stems and roots of four-week-old *in vitro*-grown plants cultured on LS medium with 3% sucrose; (b) leaves of plants grown on LS medium supplemented with 1% and 5% sucrose; and (c) leaves of plants cultivated in the greenhouse. All samples were pooled over three to six replicated plants. Isoelectric focusing was performed according to the manufacturer's instructions on Immobiline DryStrips (length 24 cm; GE Healthcare Life Sciences, Piscataway, USA) successively for narrowing ranges of pI (3–10, 4–7 and 5–6). After equilibration, the strips were transferred onto SDS denaturing polyacrylamide (12.5%) Ettan DALT II gels (25.5 × 19.5 cm; GE Healthcare), and proteins were separated as described in the Ettan DALT II User Manual. For comparative analysis, at least three replicates of every gel were silver-stained [3] and analyzed in ImageMaster 2D software version 3.10 (GE Healthcare). Coomassie Brilliant Blue R250 staining [44] was used for proteins to be subjected to subsequent analysis by MALDI-TOF MS fingerprinting after trypsin digestion (carried out at the Protein Service Laboratory, Institute for Molecular Biology and Biophysics, Zürich, Switzerland). Peptide mass lists were calibrated using Peak Erazor  and contaminating peaks were discarded, and the calibrated spectra used as a query to search EST database by Protein Prospector . Theoretical isoelectric points and molecular weights of all identified proteins were in agreement with that of corresponding protein spots. The protein sequences were covered by the matching peptides by at least 45%.

### Carbohydrate analysis

Samples (50–100 mg fresh weight) were harvested as a mixture of leaves or stems from three to eight *in vitro*-grown plants in the middle of the light period. For soil-grown plants, samples were harvested from three plants and contained pieces of fully developed leaves (third from the shoot apex). The samples were freeze-dried, boiled in 80% methanol at 75°C for 15 min, the solvent was then evaporated and the residue was dissolved in Milli-Q ultrapure water (Millipore, Bedford, USA). Thereafter, the samples were purified by centrifugation and filtration. The content of non-structural soluble carbohydrates was determined using high-performance liquid chromatography (HPLC; flow rate 0.5 ml min^-1^, temperature 80°C; sugar standards from Sigma-Aldrich) with refractometric detection (refractive index range 1–1,75; refractometer Shodex RI-71; Spectra Physics – Newport Corporation, Irvine, USA), pre-column: Hema-Bio 1000 Q+SB (Watrex, Prague, Czech Republic); column: Hi-Plex Ca^2+ ^(Polymer Laboratories, Shropshire, UK), digital output to PC – CSW 1.7 software (Data Apex, Prague, Czech Republic). For details see [43].

The starch in pellets remaining after the extraction of soluble carbohydrates was hydrolyzed by α-amylase and amyloglucosidase, and the glucose content was measured by the HPLC. For details see [40].

### Statistical analysis

Mean differences in tuber yield, average stolon length and sugar or starch content between Lada and ST plants were analyzed for statistical significances using the paired t-test at *P *< 0.01–0.05 (NCSS 2007 software; NCSS, Kaysville, USA).

## Abbreviations

MALDI-TOF MS: matrix-assisted laser desorption/ionization time of flight mass spectrometry; MSP: manganese stabilizing protein; ST mutant: spontaneously tuberizing potato mutant line;

## Authors' contributions

LF conceived the study, carried out the molecular (DNA and proteomic) analyses and most of the phenotypic characterizations of ST and Lada plants, and drafted the manuscript. HL carried out carbohydrate analysis and participated in phenotypic characterizations and in writing the paper. JFH participated in the design of proteomic analysis and the manuscript writing. ZO participated in designing the study and helped to draft the manuscript. All authors read and approved the final manuscript.
